# Site-specific phosphorylation regulates the functions of kindlin-3 in a variety of cells

**DOI:** 10.26508/lsa.201900594

**Published:** 2020-02-05

**Authors:** Katarzyna Bialkowska, Khalid Sossey-Alaoui, Elzbieta Pluskota, Lahoucine Izem, Jun Qin, Edward F Plow

**Affiliations:** 1 Department of Cardiovascular and Metabolic Sciences, Lerner Research Institute Cleveland Clinic, Cleveland, OH, USA; 2 Department of Molecular Medicine, School of Medicine, Case Western Reserve University, Cleveland, OH, USA

## Abstract

Studies of isolated cells, mice, and humans have demonstrated the vital role of the FERM domain protein kindlin-3 in integrin activation in certain hematopoietic and non-hematopoietic cells, consequent to binding to integrin β-subunits. To explore regulatory mechanisms, we developed a monoclonal antibody that selectively recognizes the phosphorylated form of Ser^484^ (pS^484^) in kindlin-3. Activation of platelets, HEL megakaryocytic-like cells and BT549 breast cancer cells led to enhanced expression of pS^484^ as assessed by immunofluorescence or Western blotting. In platelets, pS^484^ rose rapidly and transiently upon stimulation. When a mutant form of kindlin-3, T^482^S^484^/AA kindlin-3, was transduced into mouse megakaryocytes, it failed to support activation of integrin α_IIb_β_3_, whereas wild-type kindlin-3 did. In MDA-MB231 breast cancer cells, expression of T^482^S^484^/AA kindlin-3 suppressed cell spreading, migration, invasion, and VEGF production. Wild-type kindlin-3 expressing cells markedly increased tumor growth in vivo, whereas T^482^S^484^/AA kindlin-3 significantly blunted tumor progression. Thus, our data establish that a unique phosphorylation event in kindlin-3 regulates its cellular functions.

## Abstract (precis)

A new monoclonal antibody in combination with mutational analyses shows that a single serine phosphorylation in kindlin-3 is functionally important in both hematopoietic and non-hematopoietic cells.

## Introduction

The major functions of integrin adhesion receptors, ligand binding and subsequent intracellular signaling, are tightly regulated, especially for the integrins expressed on blood cells, which are exposed to a variety of circulating ligands. The integrins on these cell surfaces exist in a resting state, which preclude them from binding their ligands with high avidity/affinity. However, when cells are exposed to a stimulatory agonist, the integrins rapidly convert to active conformation, which supports robust ligand binding (Marguerie et al, 1979; Qin et al, 2004; Ma et al, 2007; Sun et al, 2019). Engagement of binding partners by the integrin cytoplasmic tails (CTs) regulates integrin activation; these binding partners induce inside-out signals that perturb the integrin transmembrane domains and then regulates the ligand binding site within extracellular region of integrin. Ligand binding can, in turn, induce outside-in signals that result in multiple cellular responses.

The kindlin family of intracellular proteins have been shown to regulate bidirectional integrin signaling. Kindlins are present in integrin-containing adhesion sites and provide a link between integrin-induced signaling and the actin cytoskeleton (Tu et al, 2003; Ussar et al, 2006; Shi et al, 2007). Three kindlin family members are present in mammals; each kindlin consists of a FERM (F for 4.1 protein, E for ezrin, R for radixin and M for moesin) domain with an inserted pleckstrin homology domain. When compared with other FERM domains, the FERM domain of kindlins shows closest homology to that of talin, another protein engaged in integrin-induced signaling (Calderwood et al, 1999; Vinogradova et al, 2002; Garcia-Alvarez et al, 2003; Tadokoro et al, 2003; Klapholz & Brown, 2017; Gough & Goult, 2018; Sun et al, 2019). Kindlins and talin act in cooperation to optimize integrin activation, by binding to integrin cytoplasmic tails, and this interaction involves their F3 (PTB) subdomains within their FERM domains (Shi et al, 2007; Ma et al, 2008; Montanez et al, 2008). Hence, cells or mice with decreased kindlin expression levels fail to activate their integrins properly. Kindlin-1 is expressed predominantly in epithelial cells; mutation of kindlin-1 in humans manifests as Kindler syndrome, a rare disease characterized by skin blistering and poikiloderma with frequent intestinal complications (Jobard et al, 2003; Siegel et al, 2003). Kindlin-2 is expressed in variety of tissues and cell types; kindlin-2 knockout in mice and zebrafish is lethal early in embryonic development (Dowling et al, 2008; Montanez et al, 2008). Postnatal loss of kindlin-2 in cardiomyocytes leads to progressive heart failure (Zhang et al, 2016). Kindlin-3–null mice show pronounced defects in platelet and leukocyte integrin-dependent functions, and kindlin-3–null mice die on day 7 postnatally (Moser et al, 2008). Humans with kindlin-3 mutations or deficiency exhibit rare syndrome referred to as LADIII. LADIII syndrome is a consequence of an inability of the cells to activate β_1_, β_2_, and β_3_ integrins, with manifestations that include susceptibility to infections, episodic bleeding, and osteopetrosis. Abnormal red blood cell shapes were also observed in some patients with LADIII (Kuijpers et al, 2009; Malinin et al, 2009; Svensson et al, 2009; Meller et al, 2012). Kindlin-3 has been shown to be present and functional in endothelial cells (Bialkowska et al, 2010) and it acts as a tumor promoter in breast cancer (BC) cells (Sossey-Alaoui et al, 2014). Despite the large body of evidence that emphasize the role of kindlin-3 in integrin-induced signaling in many different cell types, the mechanisms of kindlin-3 induced integrin activation are not well understood. One well established binding site in kindlin-3 is present in its F3 domain; this integrin-binding site is located at kindlin-3 Q^597^/W^598^. Unlike kindlin-3 knockout mice, mice in which QW have been mutated to alanines (kindlin-3 QW/AA) are viable for at least 6 mo, although they bleed excessively upon tail resection and have prolonged arterial occlusion times upon vascular injury (Xu et al, 2014).

Because the activation of integrins on blood cells requires strict regulation, it would seem that involvement of kindlin-3 could be a checkpoint in the activation process. We first considered whether talin, which is known to undergo unmasking to bind in integrin (Goksoy et al, 2008), might regulate kindlin binding to the β3 CT, but their interaction with the CT seemed to be independent (Bledzka et al, 2012) and might require a bridging protein (Gao et al, 2017; Klapproth et al, 2019). We then considered whether a posttranslational modification(s) could regulate kindlin-3 function. Indeed, in a prior study, we used mass spectrometry to show that phosphorylation of kindlin-3 occurs in platelets upon agonist stimulation in a region that is not conserved in the other two kindlin family members (Bialkowska et al, 2015). To determine whether this phosphorylation event is significant in kindlin-3 required the development of new reagents and approaches. We are now able to convincingly show that phosphorylation of kindlin-3 at S^484^ is regulated by agonists and occurs rapidly upon cell stimulation and that this posttranslational modification controls key functions of kindlin-3 in both hematopoietic and non-hematopoietic cells.

In a previous work, we have examined the relationship between kindlin-3 and BC in mouse models and human tissues (Sossey-Alaoui et al, 2014). Kindlin-3 overexpression in a BC cell line increased primary tumor growth and lung metastasis and mechanistically, kindlin-3–overexpressing cells displayed a marked increase in VEGF secretion, which was dependent upon enhanced expression of the Twist transcription factor (Sossey-Alaoui et al, 2014) and enhanced β_1_ integrin activation. The association of kindlin-3 with BC has been corroborated by some studies (Djaafri et al, 2014), although not all other studies (Azorin et al, 2018). In the present work, we establish that kindlin-3 phosphorylation is crucial for kindlin-3 function in BC cells and other cell types, including platelets, megakaryocytes, and human erythroleukemia (HEL) megakaryocytic-like cells.

## Results

### Development of kindlin-3 phosphoantibodies

**Figure S1.**
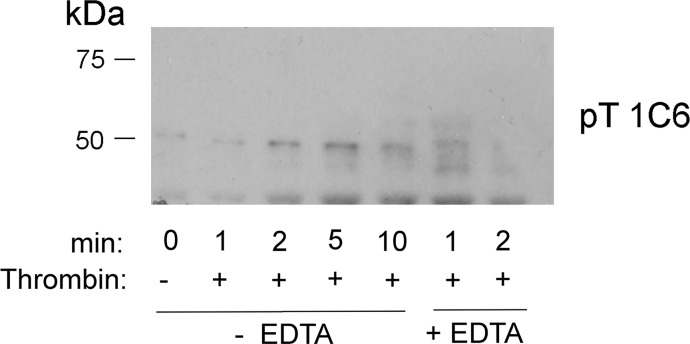
Platelet suspensions were stirred in the presence of 1 U of thrombin alone or in the presence of 10 mM EDTA for the times indicated in the absence or presence of 10 mM EDTA. Incubations were terminated by addition of 2× Laemmli sample buffer. Western blots were developed with mAb 1C6 raised to the pT^482^ peptide.

**Figure 1.**
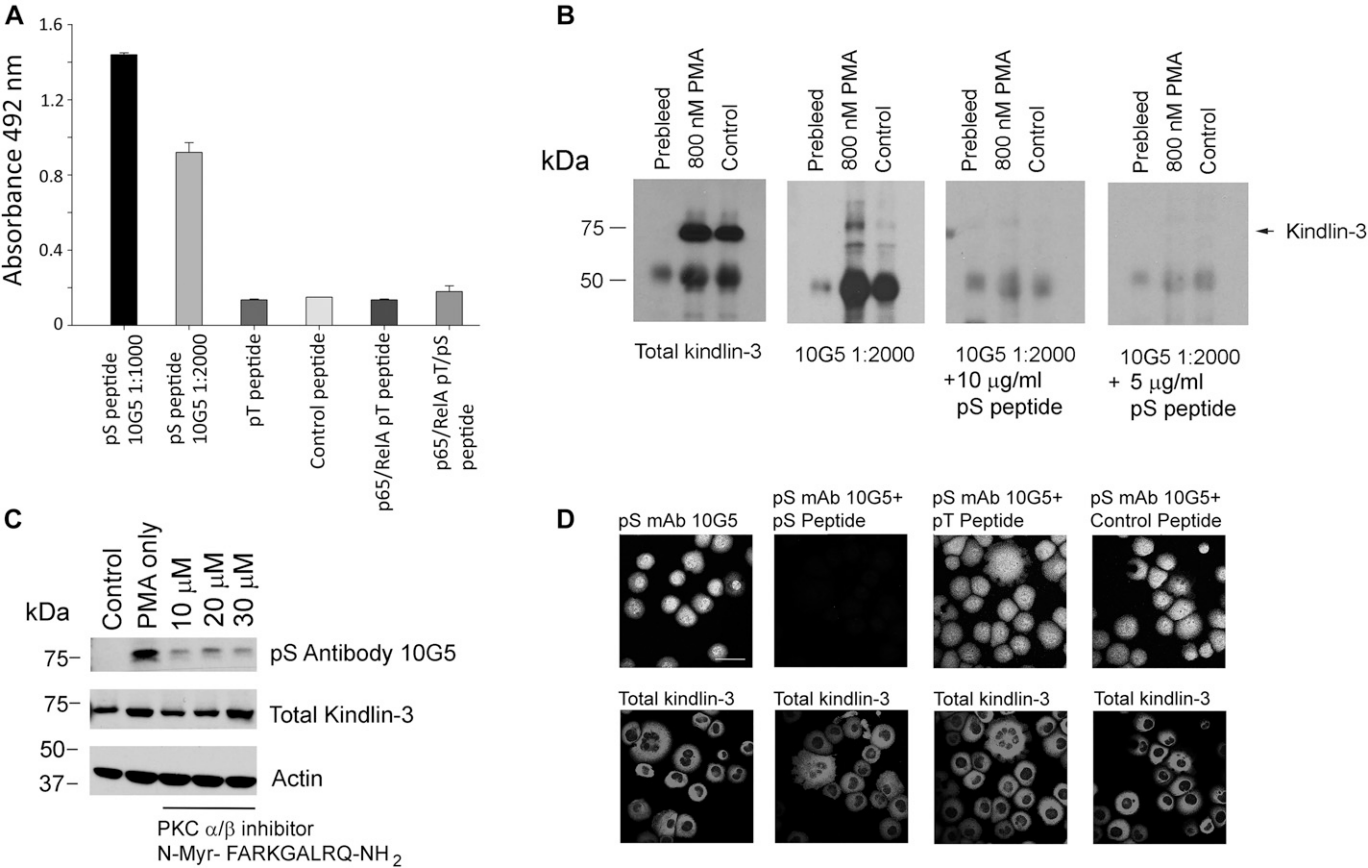
Phospho-kindlin-3–specific mAb. **(A)** mAb 10G5 to S^484^ was used in an ELISA assay. Indicated peptides were immobilized on the ELISA plate and incubated with 10G5 antibody. **(B)** Total kindlin-3 antibodies and mAb 10G5 were used to blot immunoprecipitates from control and PMA-treated human erythroleukemia (HEL) cells in the absence or presence of phosphoserine peptide. **(C)** Total kindlin-3 antibodies, actin antibodies, and mAb 10G5 were used to blot total lysates from control and PMA-treated HEL cells in the absence or presence of the PKCα/β inhibitor N-Myr- FARKGALRQ-NH_2_ at 10, 20, or 30 μM. **(D)** PMA-stimulated HEL cells were allowed to spread on immobilized fibrinogen for 60 min in the absence of serum. The cells were fixed and stained with total kindlin-3 antibodies and mAb 10G5 in the absence or presence of indicated peptides. All peptides were used at 10 μg/ml concentration. Bar, 20 μm.

Our previous work has shown that stimulation of HEL megakaryocytic cells or human platelets with agonists significantly increased kindlin-3 phosphorylation as detected by mass spectrometry (Bialkowska et al, 2015). T^482^ or S^484^ was identified as a phosphorylation site, but which of the two residues was modified could not be distinguished. These residues reside in a sequence that is unique to kindlin-3, and it is not conserved in kindlin-1 or kindlin-2. To further explore the significance of this unique posttranslational modification, we sought to generate phospho-specific antibodies to provide facile and flexible detection reagents. Two phosphopeptides of 11 amino acids (C-LSLQRTGpSGG and C-LSLQRpTGSGG) were injected as KLH conjugates into mice, and sera or hybridoma supernatants and ultimately purified IgG were obtained and tested for reactivity by ELISA on immobilized phosphopeptides and by Western blots of platelet lysates. The phosphothreonine antibody 1C6 showed specific binding to phosphothreonine peptide in ELISA (data not shown) but negligible reactivity with kindlin-3 in platelet extracts from either resting or PMA-stimulated platelets by Western blots (Fig S1) and, therefore, seemed to be of limited utility. In contrast, mAb 10G5 to pS^484^ peptide showed excellent specificity properties. In ELISA (Fig 1A), 10G5 bound specifically to the phosphoserine peptide and showed minimal reactivity with the non-phosphorylated peptide, the phosphothreonine peptide, and a control peptide containing a pS and/or a pT (Fig 1A). Next, we tested the reactivity of mAb 10G5 with HEL cells, a cell line with megakaryocytic properties, including expression of the major platelet integrin α_IIb_β_3_ that can be activated to bind ligands (Jarvinen et al, 1987; Ylanne et al, 1988, 1990; Boudignon-Proudhon et al, 1996). Control and PMA-stimulated HEL cells were immunoprecipitated with anti–kindlin-3 antibodies, and the immunoprecipitates were probed by Western blots with antibodies against total kindlin-3 and mAb 10G5 to the pS^484^ peptide (Fig 1B). Anti–kindlin-3 antibodies, but not pre-immune serum, immunoprecipitated a single ∼72-kD band (Fig 1B, total kindlin-3 blot). mAb 10G5 to pS^484^ peptide also blotted kindlin-3 in the immunoprecipitate, and this reactivity was neutralized by the presence of immunizing phosphopeptide (Fig 1B, 10G5 blots). Marked differences in reactivity between non-stimulated and PMA-stimulated HEL cells were observed with the mAb 10G5 to pS^484^ peptide (Fig 1B, 10G5, no peptide lane). These results are consistent with the induction of kindlin-3 phosphorylation upon PMA stimulation.

PKCs is a family of related serine/threonine kinases, which have been implicated in α_IIb_β_3_ integrin activation and outside-in signaling (Buensuceso et al, 2005; Soriani et al, 2006; Yacoub et al, 2006; Cifuni et al, 2008; Vilahur et al, 2018). It is well established that PKCs can be activated directly by phorbol esters, including PMA (Castagna et al, 1982; Kikkawa et al, 1983). Our previous work had suggested that PKC is involved in PMA-stimulated kindlin-3 phosphorylation using mass spectrometry to monitor the modification (Bialkowska et al, 2015). Here, HEL cells were stimulated with the PMA in the absence or presence of different concentrations of a membrane-permeable miristoylated PKC_α/β_ inhibitor, N-Myr-FARKGALRQ-NH_2_ (Ward & O’Brian, 1993), and total lysates were blotted with mAb 10G5 to pS^484^. With PMA stimulation, kindlin-3 phosphorylation as detected with 10G5 antibody was increased when compared with unstimulated cells, but the PKC_α/β_ inhibitor reduced mAb 10G5 reactivity in Western blots (Fig 1C). Based on densitometric scanning, the PKC_α/β_ inhibitor reduced the intensity of mAb 10G5 staining to 14% at 10 μM, 12% at 20 μM, and 5% at 30 μM relative to the mAb 10G5 band in cells stimulated with PMA alone. We also detected reductions in kindlin-3 phosphorylation with inhibitors for PKC_θ_ and PKC_θ/δ_ isoforms, Myr-LHQRRGAIKQAKVHHVKC-NH_2_ and 5-(3,4-Dimethoxyphenyl)-4-(1H-indol-5-ylamino)-3pyridinecarbonitrile, respectively (Osada et al, 1992; Cole et al, 2008) (data not shown), suggesting that more than one isoform of PKC might be involved directly or indirectly in kindlin-3 phosphorylation.

Further evidence for the reporter activity of mAb 10G5 to the pS^484^ peptide was derived from studies in which we stimulated HEL cells with 800 nM PMA, and the cells were allowed to spread on immobilized fibrinogen for 60 min in the absence of serum. The cells were fixed and stained with total kindlin-3 antibodies and mAb 10G5 against pS^484^ in the absence or presence of immunizing peptide (Fig 1D). Staining for total kindlin-3 was apparent and was not changed by the immunizing peptide (Fig 1D, bottom panels). Staining with mAb 10G5 was evident and was markedly diminished by phosphoserine peptide, but the phosphothreonine peptide or control and nonphosphorylated peptide had no effect (Fig 1D, upper panels).

**Figure 2.**
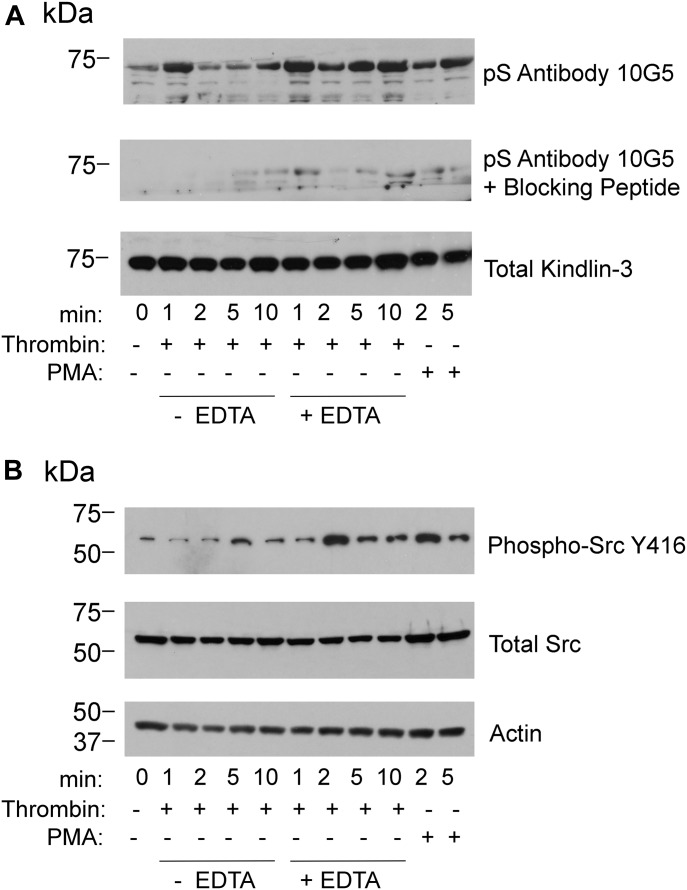
Comparison of kindlin-3 phosphorylation and Src phosphorylation in agonist-stimulated human platelets. **(A, B)** Platelet suspensions were stirred in the presence of 1 U of thrombin alone or in the presence of 10 mM EDTA or in the presence of PMA for the times indicated. Incubations were terminated by addition of 2× Laemmli sample buffer. **(A, B)** Total kindlin-3 (loading control), phosphorylated kindlin-3 (A), phosphor-Src, total Src, and actin (loading control) (B) were detected on Western blots. Phosphoserine blocking peptide was used at 10 μg/ml. The blots are representative of three independent experiments; two other experiments are shown in Fig S2.

**Figure S2.**
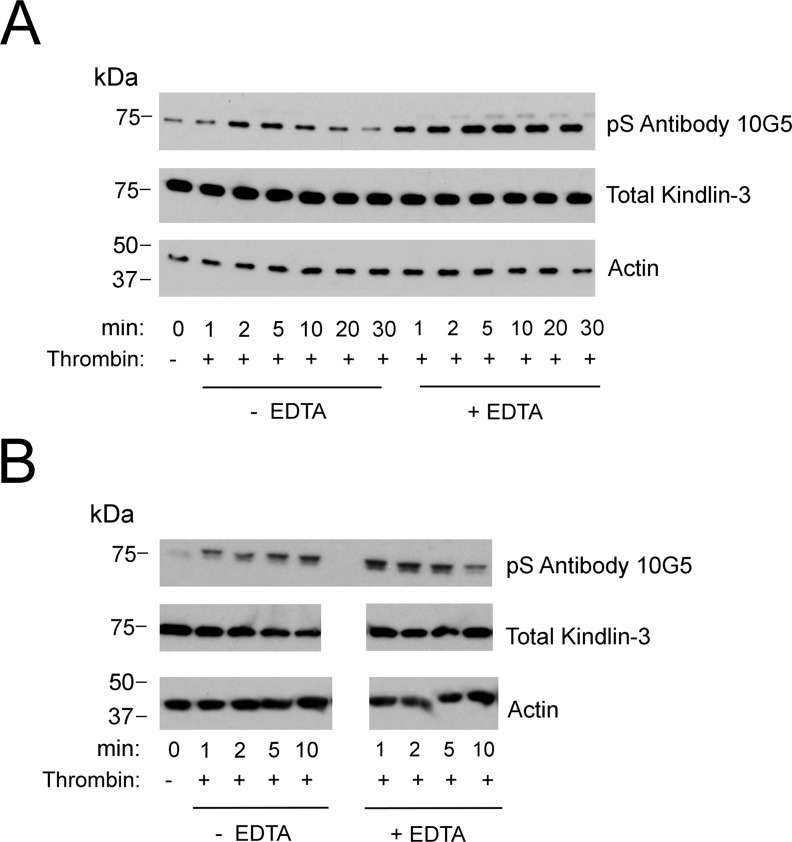
Kindlin-3 phosphorylation in agonist-stimulated human platelets. **(A, B)** Two independent experiments using platelets from different donors. Platelet suspensions were stirred in the presence of 1 U of thrombin alone or in the presence of 10 mM EDTA for the times indicated. Incubations were terminated by addition of 2× Laemmli sample buffer. Total kindlin-3 and actin (loading controls) and phosphorylated kindlin-3 were detected on Western blots.

With this evidence of appropriate specificity, we sought to use the mAb 10G5 to pS^484^ on kindlin-3 status in platelets. Kindlin-3 phosphorylation as observed by mass spectroscopy occurred in platelets upon thrombin or PMA stimulation; maximal kindlin-3 phosphorylation was observed in the presence of EDTA, which prevents platelet aggregation, but still allows integrin activation (Bialkowska et al, 2015). We used mAb 10G5 to pS^484^ to follow the time course of kindlin-3 phosphorylation in platelets stimulated with 1 U of thrombin in the absence or presence of 10 mM EDTA for 1, 2, 5, or 10 min or in the presence of 800 nM PMA for 2 and 5 min. Total platelet lysates were assessed by Western blotting with mAb 10G5. Little kindlin-3 S^484^ phosphorylation was detected in unstimulated platelets (Fig 2A, top panel, 0 min). With thrombin stimulation, kindlin-3 phosphorylation increased rapidly and was readily detected at 1 min. This level diminished over time and returned to basal levels by 5 min (Fig 2A, top panel, lanes with no EDTA). When platelets were prevented from aggregating with EDTA (Fig 2A, top panel, lanes with EDTA), kindlin-3 phosphorylation increased and was more stable. Also stimulation with 800 nM PMA increased kindlin-3 phosphorylation, but to a lesser extent than thrombin stimulation (Fig 2A, top panel, lanes with PMA). Similar patterns of kindlin-3 phosphorylation were observed in two additional experiments using platelets from different donors (Fig S2). Immunoreactivity with mAb 10G5 was markedly diminished in the presence of phosphoserine peptide (Fig 2A, middle panel), indicative of specificity. As a reference point for comparison, phosphorylation of Src on Y^416^, a hallmark of Src activation (Hunter, 1987; Shattil, 2005), also followed a transient pattern (Fig 2B, top panel), although Src phosphorylation occurred less rapidly in the presence of EDTA when compared with kindlin-3 phosphorylation on S^484^.

**Figure 3.**
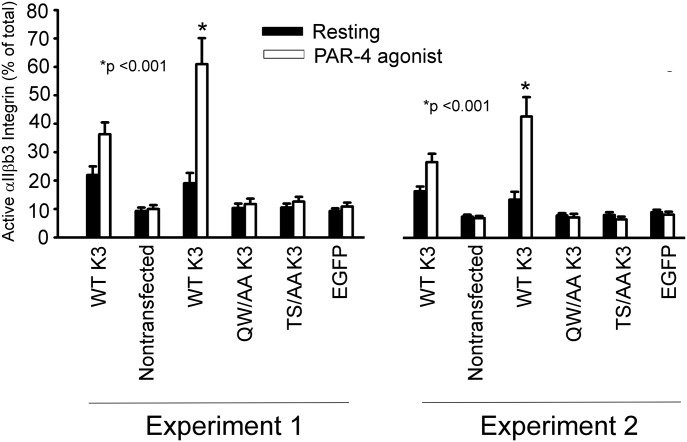
Effect of kindlin-3 variants on activation of α_IIb_β_3_ in megakaryocytes. Bone marrow megakaryocytes were isolated from mice with low expression of kindlin-3 and transduced with lentiviruses to express EGFP-WT kindlin-3, EGFP-Q^597^W^598^/AA kindlin-3, EGFP-T^482^S^484^/AA kindlin-3, or EGFP alone and were then either untreated or stimulated with PAR-4 agonist peptide. α_IIb_β_3_ integrin activation was measured by flow cytometry in EGFP-positive cell populations using PE-labeled Ab to activation-dependent epitope of α_IIb_β_3_ (clone JON/A) or total α_IIb_β_3_ with rat antimouse CD41-PerCP/Cy5.5. The data are expressed as % activation = (MFI JONA/MFI total CD41) × 100. The error bars represent means ± SE (*P < 0.001, two-tailed *t* test). The left and right data sets are from two independent experiments, with triplicates performed in each experiment.

To further assess the importance of kindlin-3 phosphorylation in regulation of α_IIb_β_3_ activation, megakaryocytes were isolated from the bone marrow of mice with low expression of kindlin-3 (∼10% of kindlin-3 levels in WT mice), which, according to Klapproth et al (2015), is sufficient to prevent spontaneous bleeding). The procedure of Shiraga et al (1999) was used to isolate megakaryocytes, which were then infected with lentivirus constructs for human EGFP-tagged WT kindlin-3, T^482^S^484^/AA kindlin-3, and Q^597^W^598^/AA kindlin-3. The double T^482^S^484^/AA kindlin-3 mutant was used to preclude phosphorylation of T^482^ if S^484^ was not available or vice versa and our previous demonstration that the T^482^S^484^/AA expressed well and retained many kindlin-3 functions (Bialkowska et al, 2015). On day 2 postinfection, α_IIb_β_3_ integrin activation was assessed on EGFP-gated population only using mAb JON/A, which is specific for activated mouse α_IIb_β_3_ integrin (Bergmeier et al, 2002), with or without PAR-4 agonist. When normalized to total α_IIb_β_3_ integrin expression, wild-type, control megakaryocytes, expressing endogenous kindlin-3 increased their integrin activation almost twofold upon agonist stimulation (Fig 3, WT kindlin-3). Megakaryocytes from the mice with low kindlin-3 failed to activate α_IIb_β_3_ integrin even upon agonist stimulation (Fig 3, non-transfected). Megakaryocytes overexpressing WT kindlin-3 increased α_IIb_β_3_ integrin activation upon PAR4 agonist stimulation as measured by JON/A binding (Fig 3, WT kindlin-3). In contrast, megakaryocytes expressing T^482^S^484^/AA kindlin-3 failed to activate α_IIb_β_3_ integrin. A similar failure to activate was observed in megakaryocytes expressing Q^597^W^598^/AA kindlin-3 or EGFP alone (Fig 3). These results were replicated in two independent experiments (Fig 3).

**Figure S3.**
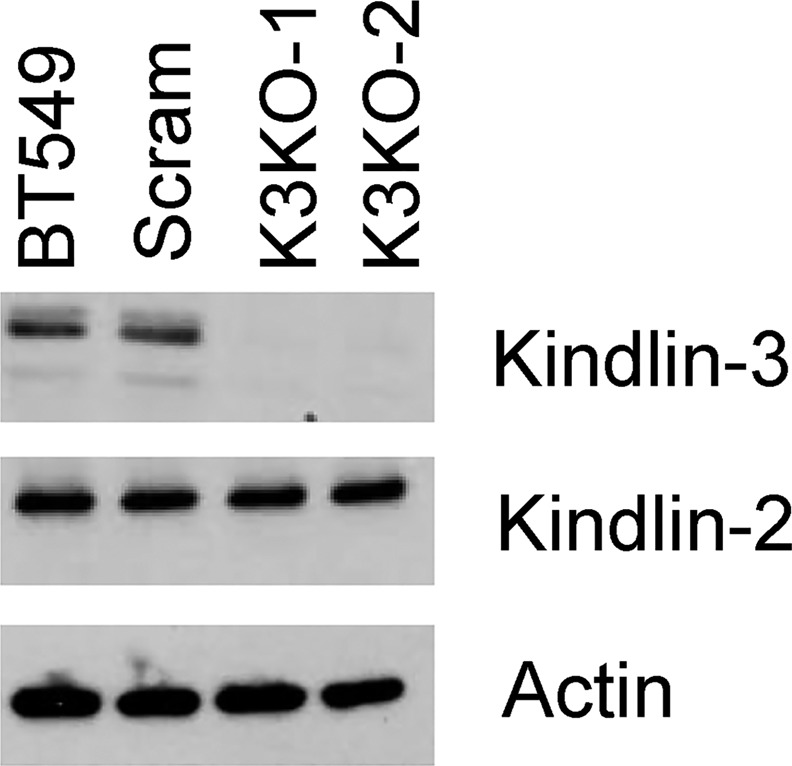
Western blots developed with anti-kindlin-3 antibody of lysates from control BT549 cells, cells transduced with a scrambled sgRNA (Scram), and cells treated with two independent sgRNAs targeting kindlin-3. Β-actin was used as a loading control.

**Figure 4.**
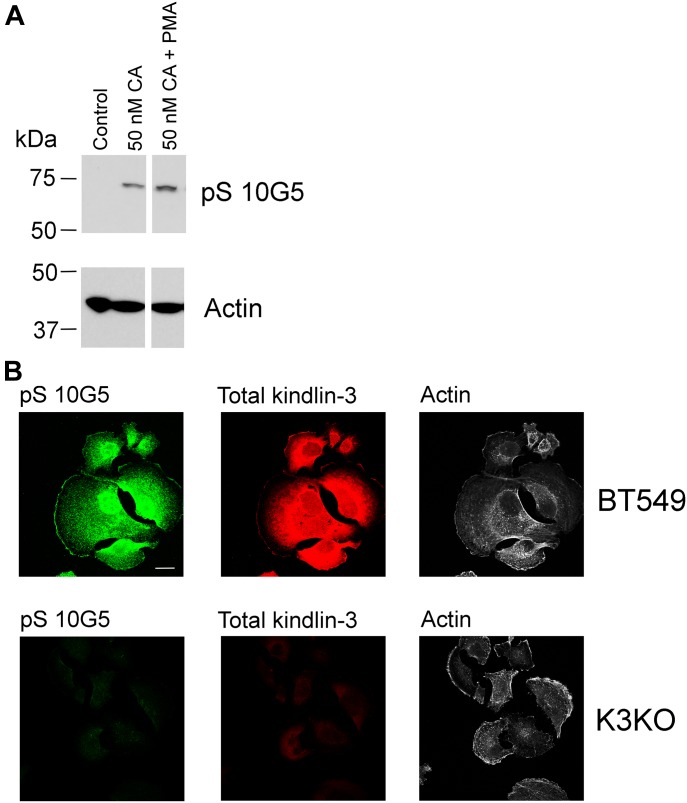
Kindlin-3 phosphorylation in BT549 cells. **(A)** BT549 cells were treated with calyculin A in the absence or presence of 800 nM PMA. Incubations were terminated by addition of 2× Laemmli sample buffer and phosphorylated kindlin-3 and actin (loading control) were detected on Western blots. The blots are representative of three independent experiments; two other experiments are shown in Fig S4. **(B)** BT549 parental cells and BT549 kindlin-3–deficient cells were allowed to spread on immobilized fibronectin for 60 min in the absence of serum. The cells were fixed and stained with total kindlin-3 antibodies and mAb 10G5 to pS^484^. Bar, 20 μm.

**Figure S4.**
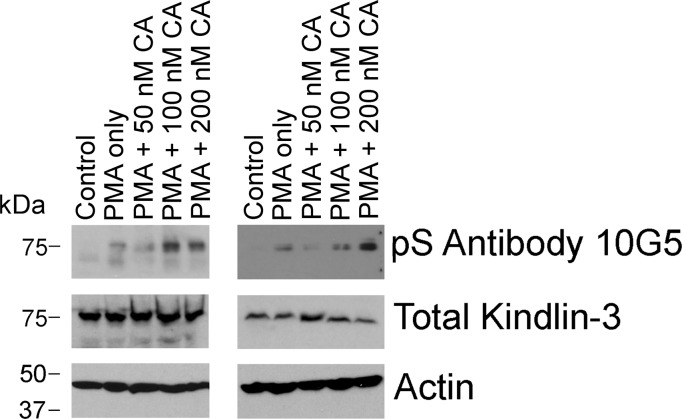
Kindlin-3 phosphorylation in BT549 cells, a triple negative breast cancer cell line. These cells were treated with 800 nM PMA and different concentrations of calyculin A. Incubations were terminated by addition of 2× Laemmli sample buffer, and phosphorylated kindlin-3 and actin (loading control) were detected on Western blots. Two independent experiments are shown.

To determine the impact of kindlin-3 phosphorylation in a non-hematopoietic cell, we tested the triple-negative BT549 BC cell line, which expresses high levels of kindlin-3 (Sossey-Alaoui et al, 2014) (Fig S3, BT549 lane) for reactivity with mAb 10G5. BT549 cells were serum-starved overnight and then treated with 50 nM calyculin A, a potent phosphatase inhibitor (Resjo et al, 1999) for 10 min in the absence or presence of 800 nM PMA, and total cell lysates were assayed by Western blotting for phosphorylated kindlin-3 with mAb 10G5. There was very little or no kindlin-3 phosphorylation of S^484^ in control, unstimulated BT549 cells (Fig 4A, lane 1). Treatment of the cells with a 50 nM calyculin A increased kindlin-3 phosphorylation rapidly (Fig 4A, line 2), and this level was further enhanced by addition of PMA (Fig 4A, lane 3). Similar patterns of kindlin-3 phosphorylation were observed in two additional independent experiments (Fig S4).

Spreading assays were also performed on parental BT549 cells that had been rendered deficient in kindlin-3 using CRISPR/Cas9 technology. As shown in Fig S2, the two sgRNAs deleted kindlin-3 but did not alter kindlin-2 expression. The cells were allowed to spread on immobilized fibronectin for 1 h and then stained with total kindlin-3 antibodies, mAb 10G5 against kindlin-3 pS^484^ and phalloidin for actin. The immunofluorescent micrographs in Fig 4B show strong staining of the parental BT549 cells for total kindlin-3 and phosphorylated kindlin-3 (Fig 4B, top panels), whereas the staining was negligible for both kindlin-3 and pS-kindlin-3 in the kindlin-3 KO BT549 cells. Thus, using three different cell types, megakaryocyte-like, platelets, and a BC cell line, kindlin-3 is shown to undergo phosphorylation, and the level of its phosphorylation was enhanced by stimulation of the cells or reducing phosphatase activity.

### Kindlin-3 phosphorylation regulates BC cell properties and tumor progression in vivo

**Figure 5.**
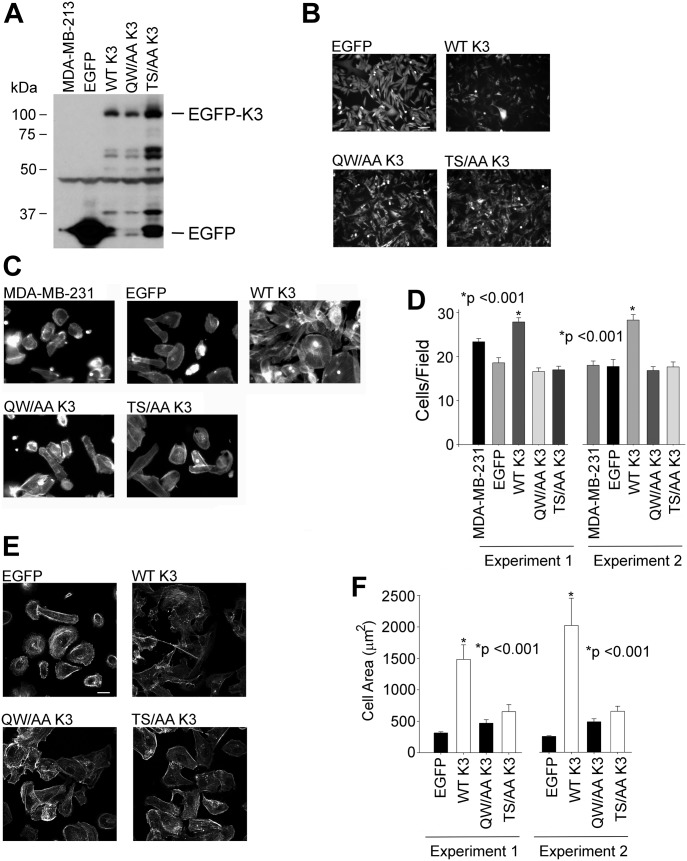
Kindlin-3–enhanced invasion and spreading of breast cancer cells in vitro is blunted in cells expressing T^482^S^484^/AA kindlin-3. **(A)** Immunoblots of total cell lysates from MDA-MB-231 cells expressing EGFP alone or EGFP-kindlin-3 constructs, probed with anti-EGFP. **(B)** Representative micrographs of EGFP-expressing and EGFP–kindlin-3–overexpressing MDA-MB-231 cells in the culture visualized with EGFP fluorescence. Bar, 100 μm. **(C)** Representative micrographs of EGFP-expressing and EGFP-kindlin-3–overexpressing MDA-MB-231 cells that were induced to invade through a Matrigel-coated membrane. The cells were visualized with Alexa-568 phalloidin to stain filamentous actin. Bar, 50 μm. **(D)** Invading cells were counted from 10 different fields and plotted as an average of the number of invading cells per field (*P < 0.001, two-tailed *t* test). The error bars represent means ± SE. The left and right data sets are from two independent experiments. **(E)** Representative micrographs of EGFP-expressing and EGFP-kindlin-3–overexpressing MDA-MB-231 cells spreading on fibronectin for 1 h. The cells were visualized with Alexa-568 phalloidin to stain filamentous actin. **(F)** The areas of cells were measured using ImageJ software, and 300 cells were quantified in each experiment (*P < 0.001, two-tailed *t* test). The error bars represent means ± SE. The left and right data sets are from two independent experiments. Bar, 10 μm.

Our previous work showed that kindlin-3 influences the growth and metastasis of triple-negative human BC lines in mice (Sossey-Alaoui et al, 2014). To determine if kindlin-3 phosphorylation influences tumorogenic properties, we used MDA-MB-231 cells, which express relatively low levels of kindlin-3, to overexpress kindlin-3 mutants. cDNAs for WT-kindlin-3, a T^482^S^484^/AA kindlin-3 or a kindlin-3 Q^597^/W^598^/AA, which disables its primary integrin-binding site, was overexpressed in these cells as EGFP fusions. Stable expression of the kindlin-3 variants or EGFP-alone was monitored by Western blots (Fig 5A) and EGFP fluorescence (Fig 5B). We first assessed the properties of these cells in in vitro analyses. In Boyden chamber invasion assays, significantly more kindlin-3–overexpressing cells traversed the Matrigel-coated inserts and invaded the lower chamber as compared with the cells expressing EGFP alone (Fig 5C and D). This response is consistent with that previously observed with stably transfected MDA-MB-231 cells (Sossey-Alaoui et al, 2014), indicating that kindlin-3 confers enhanced migratory/invasive advantage to these BC cells. The enhanced invasion of the kindlin-3–overexpressing MDA-MB-231 cells was significantly blunted in cells not only expressing kindlin-3 Q^597^W^598^/AA but also kindlin-3 T^482^S^484^/AA, which emphasizes the importance of kindlin-3 phosphorylation in the invasive properties of these cancer cells.

We next assessed the capacity of the T^482^S^484^/AA kindlin-3 mutant in integrin-mediated cell spreading. The transfected MDA-MB-231 cells were plated onto fibronectin for 30 min, they were fixed and actin visualized with Alexa-568 phalloidin, and the cell area was determined by measuring the area of 100 cells for each of the constructs. Compared with control EGFP-expressing cells, the cells transfected with WT kindlin-3 exhibited markedly enhanced spreading (Fig 5E). In contrast, T^482^S^484^/AA kindlin-3–expressing cells had areas similar to EGFP-expressing cells, which were also similar to the effect of Q^597^W^598^/AA kindlin-3, observations consistent with the importance of phosphorylation in integrin-induced spreading of these BC cells (Fig 5F). These results were replicated in two independent experiments (Fig 5F).

**Figure 6.**
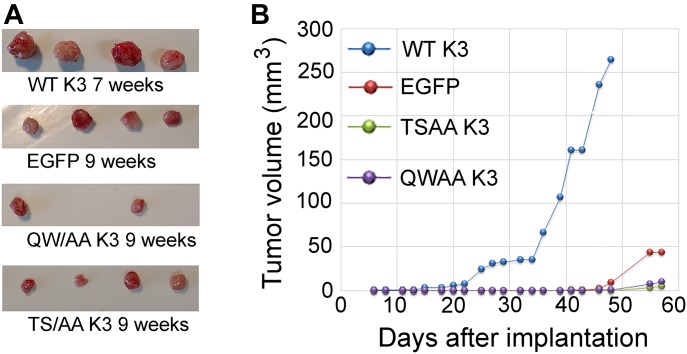
WT kindlin-3 enhances primary tumor growth of breast cancer in vivo in mouse models, and tumor growth is blunted for cells expressing T^482^S^484^/AA kindlin-3. **(A)** Tumors generated from inoculation of EGFP or EGFP–kindlin-3–expressing MDA-MB-231 cells into the mammary fat pads of NSG mice. **(B)** Tumor growth curves for EGFP-expressing and EGFP–kindlin-3–overexpressing MDA-MB-231 cells. Data points represent means ± SE of tumor volume.

To evaluate the effects of kindlin-3 phosphorylation in an in vivo setting, we performed tumor growth assays in mice. MDA-MB-231 cells expressing the kindlin-3 variants were implanted in the mammary fat pads of NSG mice, and tumor growth was assessed over 7–9 wk. Although every mouse in the EGFP-alone and WT kindlin-3 groups developed tumors (100% tumor incidence) after a 4-wk latency period, the tumor burden was significantly higher in the mice implanted with the kindlin-3–overexpressing cells. Tumors in mice implanted with cells expressing T^482^S^484^/AA kindlin-3 were significantly smaller than those overexpressing WT kindlin-3 and also slightly smaller than tumors expressing EGFP alone (Fig 6A and B). Only two tumors expressing Q^597^W^598^/AA kindlin-3 developed over a 9-wk period (Fig 6A), and these tumors were significantly smaller that tumors overexpressing WT kindlin-3 (Fig 6A and B). Thus, WT kindlin-3 enhances the rate of tumor growth in vivo, and this effect is dependent on kindlin-3 phosphorylation as well as integrin binding.

**Figure 7.**
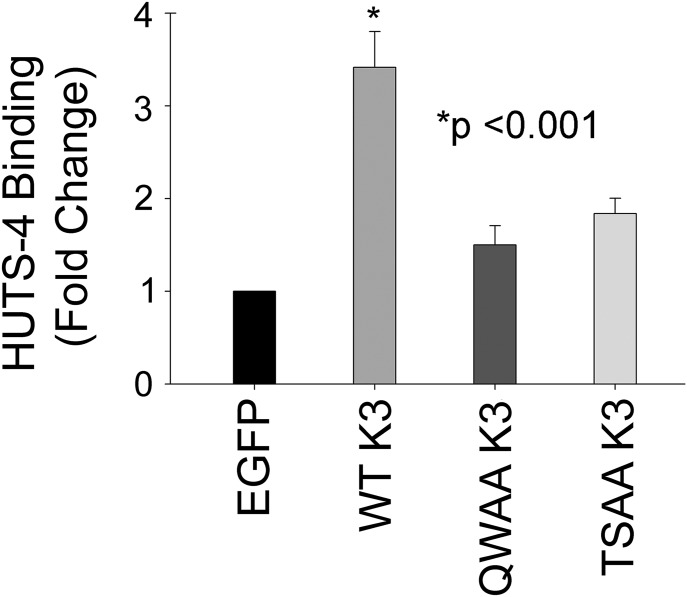
Quantification of cell surface β_1_-integrin activation in MDA-MB-231 cells. Activation was measured in the EGFP-expressing and EGFP–kindlin-3–overexpressing MDA-MB-231 cells using HUTS4 antibody binding, normalized to total β_1_-integrin expression levels, and plotted as a fold change to the EGFP-expressing cells. The error bars represent means ± SE of three independent experiments (*P < 0.001, two-tailed *t* test).

With the profound effect of kindlin-3 phosphorylation on tumor growth, we examined some of the molecular events known to be kindlin-3-dependent. First, we considered the expression and activation of integrins on the surface of the MDA-MB-231 transfectants. We had previously reported that both total surface expression (measured with monoclonal antibody mAb 13) and activation of β1-integrin (measured with HUTS-4, an antibody specific for activated β_1_ integrins (Luque et al, 1996) increased in MDA-MB-231 cells overexpressing WT kindlin-3 versus EGFP controls as assessed by flow cytometry (Sossey-Alaoui et al, 2014). When normalized to total β1 integrin expression, WT kindlin-3–overexpressing cells increased β_1_ integrin activation measured by HUTS-4 binding (Fig 7). In contrast, integrin activation in cells expressing T^482^S^484^/AA kindlin-3 was similar to activation in EGFP-expressing cells. Cells expressing the Q^597^W^598^/AA kindlin-3 also showed minimal integrin activation (Fig 7).

**Figure 8.**
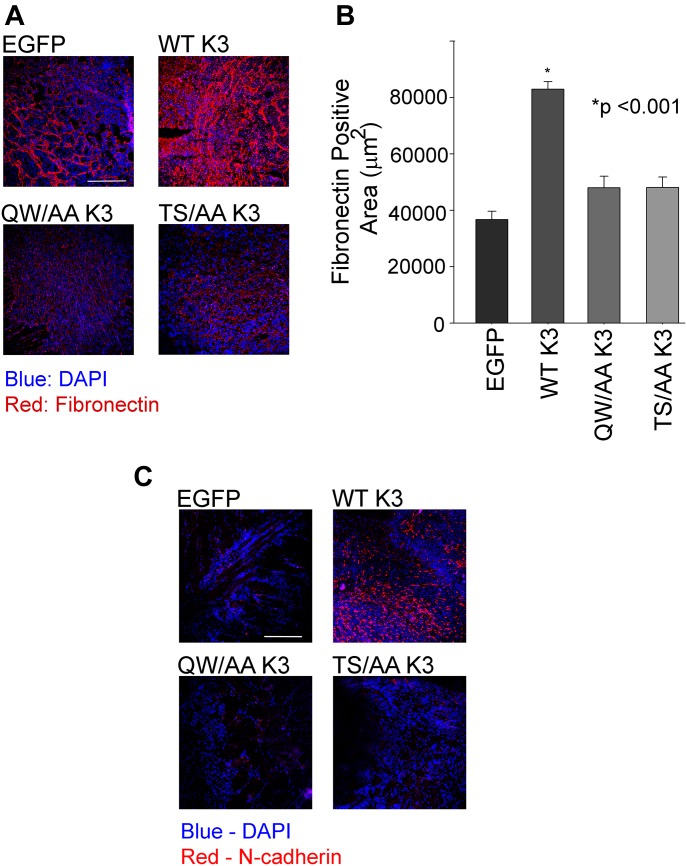
Kindlin-3–mediated regulation of the epithelial-to-mesenchymal transition program in breast cancer depends on kindlin-3 phosphorylation. **(A)** Representative immunofluorescence confocal micrographs of sections of mammary fat pad tumors derived from EGFP-expressing and EGFP–kindlin-3–overexpressing MDA-MB-231 cells, stained with anti-fibronectin (red fluorescence). Cell nuclei were counterstained with DAPI. Bar, 200 μm. **(B)** Quantification of fibronectin area in the tumors described in (B), as determined by the average fibronectin-positive area per tumor section. The fibronectin-positive areas were measured using ImageJ software, and 20 fields were quantified per group (*P < 0.001, two-tailed *t* test). **(C)** Representative immunofluorescence confocal micrographs of sections of mammary fat pad tumors derived from EGFP-expressing and EGFP–kindlin-3–overexpressing MDA-MB-231 cells, stained with anti–N-cadherin (red fluorescence). Cell nuclei were counterstained with DAPI. Bar, 200 μm.

One of the major characteristic of the neoplastic transformation is epithelial-to-mesenchymal transition (EMT), which is characterized by acquisition of high mobility and a fibroblastoid apolar phenotype. One of the hallmarks of EMT is the up-regulation of specific markers, including N-cadherin and fibronectin. Cells expressing WT kindlin-3 enhanced EMT markers expression and enhanced fibronectin deposition (Fig 8A and B). Tumors derived from WT kindlin-3–overexpressing cells show increased N-cadherin expression (Fig 8C), when compared with tumor derived from EGFP-expressing cells. Fibronectin deposition and N-cadherin expression (Qu et al, 2014) were significantly blunted in tumors derived from T^482^S^484^/AA kindlin-3–expressing cells (Fig 8A–C), emphasizing the importance of kindlin-3 phosphosite in enhancing the expression of EMT markers.

**Figure 9.**
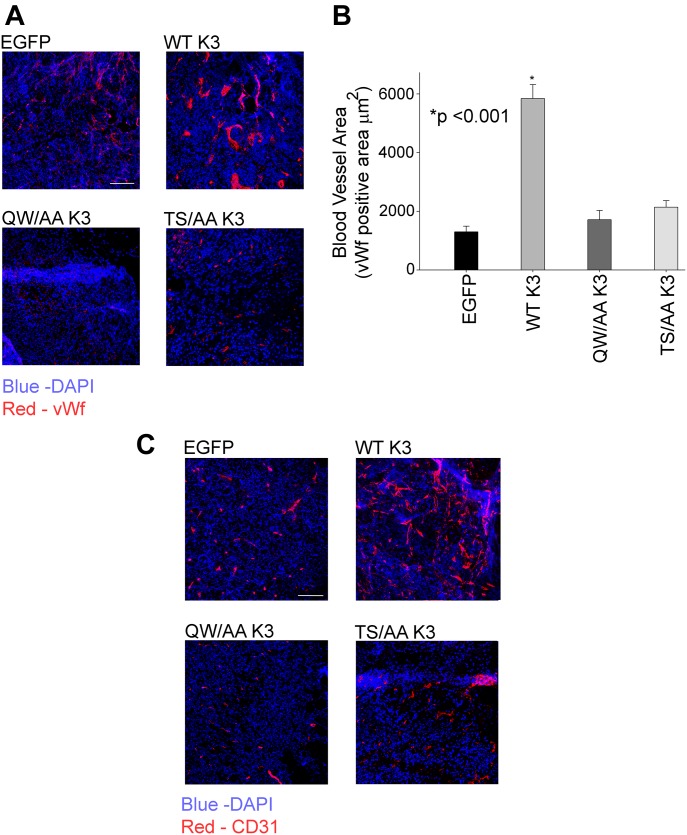
Angiogenesis is inhibited in tumors formed by cells expressing T^482^S^484^/AA kindlin-3. **(A)** Representative immunofluorescence confocal micrographs of sections of mammary fat pad tumors from mice implanted with EGFP–kindlin-3 or EGFP-expressing MDA-MB-231 cells stained with anti-vWf (red) to detect tumor-associated blood vessels. Cell nuclei were counterstained with DAPI (blue). Bar, 200 μm. **(B)** Quantification of angiogenesis from the EGFP and kindlin-3 groups, as determined by the average blood vessel area per tumor section. The areas of vessels were measured using ImageJ software, and 20 fields were quantified per group (*P < 0.001, two-tailed *t* test). **(C)** Representative immunofluorescence confocal micrographs of sections of mammary fat pad tumors from mice implanted with EGFP–kindlin-3–overexpressing MDA-MB-231 or EGFP-expressing and cells stained with anti-CD31 (red) to detect tumor-associated blood vessels. Cell nuclei were counterstained with DAPI (blue). Bar, 200 μm.

Angiogenesis is critical for tumor growth; hence, we determined whether kindlin-3 phosphorylation affected tumor microvasculature. IHC using antibody to Von Willebrand factor (vWf) and CD31, endothelial cell markers, revealed a significant increase in the number and size, as well as a dismorphic architecture of blood vessels in tumors from WT kindlin-3–overexpressing compared with EGFP-expressing cells (Fig 9A–C), as reported previously (Sossey-Alaoui et al, 2014). The numbers and size of the blood vessels from tumors expressing T^482^S^484^/AA kindlin-3 were significantly decreased when compared with tumors expressing WT kindlin-3 and similar to those from EGFP-expressing and the tumors and the Q^597^W^598^/AA kindlin-3 (Fig 9A–C).

**Figure 10.**
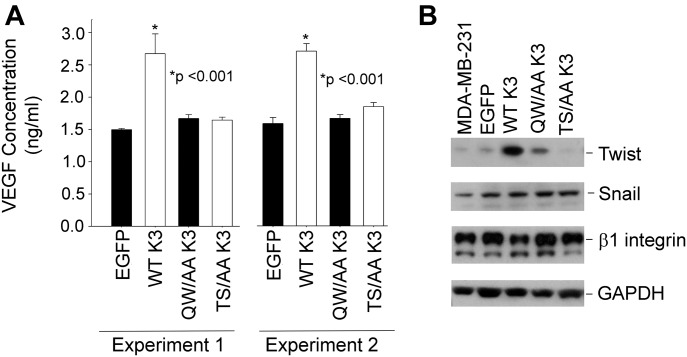
Kindlin-3 enhanced VEGF secretion and Twist expression is blunted in cells expressing T^^482^^S^^484^^/AA kindlin-3. (A) Quantification of secreted VEGF-A in the conditioned media of EGFP-expressing and EGFP–kindlin-3–overexpressing MDA-MB-231 cells using ELISA. MDA-MB-231 cells were cultured in serum-free DMEM medium for 24 h before experiment. The error bars represent means ± SE (*P < 0.001, two-tailed *t* test). The left and right data sets are from two independent experiments, with triplicates performed in each experiment. **(B)** Immunoblotting with the indicated antibodies of cell lysates from EGFP-expressing and EGFP–kindlin-3–expressing MDA-MB-231.

Tumor angiogenesis is highly dependent on secretion of angiogenic growth factor VEGF by tumor cells, and kindlins have been shown to influence VEGF production (Sossey-Alaoui et al, 2014). This effect of kindlin-3 is shown with the MDA-MB-231 cell lines as WT kindlin-3–overexpressing cells increased their release of VEGF by almost threefold compared with the EGFP-control cells. In contrast, T^482^S^484^/AA kindlin-3 cells exhibited a minimal increase in VEGF release and similar to the level released from the Q^597^W^598^/AA kindlin-3 cells (Fig 10A). These results were replicated in two independent experiments (Fig 10A). The transcription factor Twist has been shown to be a master regulator of cancer metastasis by promoting tumor angiogenesis (Mironchik et al, 2005). In cells overexpressing WT kindlin-3, Twist protein expression was elevated compared with cells expressing control EGFP. As shown in Fig 10B, Twist expression was readily detected in cells overexpressing WT kindlin-3. However, little or no Twist could be detected not only in the cells expressing Q^597^W^598^/AA but also in the cells expressing T^482^S^484^/AA kindlin-3 (Fig 10B). As a control, the levels of another transcription factor, Snail, remained unchanged regardless of the form of kindlin-3 expressed (Fig 10B) in the cells.

## Discussion

Kindlins play an indispensable role in integrin activation in many hematopoietic and non-hematopoietic cells, but the mechanisms by which they impact integrin activation remain largely unresolved. Phosphorylation is emerging to be such a regulatory mechanism. Kindlin-2 phosphorylation on Y^193^ by Src and kindlin-2-Src interaction regulates integrin outside-in signaling and controls cell migration and proliferation (Qu et al, 2014). Reciprocally, kindlin-2 Y^193^ phosphorylation activates and maintains Src kinase activity (Liu et al, 2015). Calpain cleaves kindlin-3 at Y^373^ in response to cell stimulation and/or an increase in intracellular calcium (Zhao et al, 2012), which in turn regulates the dynamics of the interaction between β integrin subunit and kindlin-3. In our previous study (Bialkowska et al, 2015), we identified agonist-induced kindlin-3 phosphorylation on T^482^ and/or S^484^ in HEL cells and platelets. The identified phosphosite resides in highly variable region of kindlin-3 and at least one of the two residues, T^482^ or S^484^, is evolutionarily conserved in all kindlin-3 orthologues analyzed, including mouse, bovine, and zebrafish kindlin-3.

In this study, we developed mAbs against phosphorylated kindlin-3 and showed that 10G5 clone specifically recognizes pS^484^ in different cell types, including platelets, megakaryocytic cells and BC cells, and this phosphorylation was enhanced by agonist stimulation. The experimental usefulness of phosphospecific antibodies is limited by the extent to which they exhibit specificity. For mAb 10G5, specificity for kindlin-3 pS^484^ was demonstrated by the following: 1) neutralization of immunoblot reactivity in the presence of specific phosphopeptide in HEL cells and platelets (Figs 1A and 2A); 2) immunoreactivity in HEL cells spreading on integrin substrate, which diminished in the presence of specific phosphopeptide but not in the presence of non-phosphorylated peptides (Fig 1B); 3) increase in immunoblot reactivity in the presence of calyculin A and PMA treatment in BT549 BC cell line (Fig 4A); 4) and the lack of reactivity in BT549 cells lacking kindlin-3 expression (Fig 4B).

In our previous work, we used mass spectrometry to show that thrombin induced phosphorylation of kindlin-3. It appeared that the phosphorylation of kindlin-3 was followed by rapid dephosphorylation in aggregating platelets (Bialkowska et al, 2015). Here, phosphospecific antibody allowed us to not only confirm but to extend on these observations. As disclosed by the pS^484^ mAb, kindlin-3 phosphorylation was rapid, clearly detected within 1 min after thrombin stimulation, and the phosphorylation diminished by 5 min in the absence of EDTA. In the presence of EDTA, which prevented the platelets from aggregating, the increase in kindlin-3 phosphorylation was sustained for at least 10 min when platelets were present. This pattern is not unusual for platelet proteins, which undergo a myriad of rapid phosphorylation/dephosphorylation reactions in response to various platelet agonists (Zimman et al, 2014; Beck et al, 2017). For time course experiments, we choose to compare kindlin-3 phosphorylation with Src, although the Scr phosphorylation was monitored on a tyrosine, pY^464^, as contrasted in a serine, pS^484^, of kindlin-3. Impetus for this comparison resides in the fact that both kindlin and Src have been implicated in outside-in signaling (Shattil, 2005; Feng et al, 2012; Xue et al, 2013; Liao et al, 2015; Bledzka et al, 2016) and, importantly, both bind to the extreme C-terminal end of β_3_ integrin. Recent data from Liao et al (2015) suggests that kindlin-3 is the preferred binding partner, and its phosphorylation does seem to be slightly more rapid. Phosphorylation of kindlin-3 does not appear to prevent its binding to β_3_ integrin (Bialkowska et al, 2015). However, the role of kindlin-3 in outside-in signaling does not exclude a role in inside-out signaling as well (Ye et al, 2013). The failure of the mutant T^482^S^484^/AA kindlin-3 to support cell spreading would be consistent with such a role.

The mechanism by which phosphorylation activates kindlin-3 remains to be determined. This posttranslational modification does appear to be PKC dependent based on the effects of specific inhibitors and consistent with our previous findings (Bialkowska et al, 2015). Our inhibitor studies implicate PKC_α/β_ in the phosphorylation process, but PKC_δ/θ_ may also be involved in a network leading to the posttranslational modification itself or the extent of the modification. Because S^484^ resides in a region that is not conserved among the kindlins, the crystal structure of kindlin-2 (Li et al, 2017) is not insightful. Molecular modeling of kindlin-3 using the ROBETTA program (Kim et al, 2004) suggested that T^482^S^484^ of kindlin-3 resides at the hydrated surface. In this orientation, phosphorylation could support binding of a phosphor-specific partners. However, it is unclear if any of the reported binding partners of kindlins (Plow et al, 2016) interacts uniquely with kindlin-3. Alternatively, it has been reported that dimerization of kindlin-2 regulates its integrin activation function (Li et al, 2017) and phosphorylation could regulate the association between kindlin-3 molecules. These possibilities remain to be investigated in future studies.

Although kindlin-3 is highly expressed in the hematopoietic system, there are only limited reports about its involvement in blood cell cancers, and a role of kindlin-3 phosphorylation in cancer cell is not known. Kindlin-3 was found to be associated with both acute and chronic myeloid leukemias (Wu et al, 2012; Qu et al, 2015). Kindlin-3 may influence proliferation of human chronic myeloid leukemia K562 cells through its regulation of c-Myc protein expression and kindlin-3 controlled the growth of these cells in a xenograft model (Qu et al, 2015). Recent studies have identified kindlin-3 in solid tumors, but its role is controversial. A study by Djaafri et al (2014) failed to detect kindlin-3 in BC cells and concluded that kindlin-3 has tumor suppressor function when expressed MDA-MB-231 BC cells. However, independent evidence for the role of kindlin-3 in BC can be taken from interrogation of the Oncomine database (http://www.oncomine.com) in which kindlin-3 ranked in the top 3% of up-regulated genes. Furthermore, in glioblastoma, kindlin-3 contributed to resistance to telozolomide and cell proliferation through Wnt signaling (Lu et al, 2017), whereas overexpression of kindlin-3 in melanoma cells led to imbalanced Rho GTP-ase activation and inhibition of cell migration (Feng et al, 2017).

In the present work, we found that kindlin-3 phosphorylation was a tumor promotor. We establish that kindlin-3 phosphorylation is crucial for kindlin-3 function in BC as supported by the following findings: kindlin-3 induced spreading and invasion in vitro and tumor progression, and metastasis in mice in vivo is blunted in T^482^S^484^/AA kindlin-3 cells; kindlin-3 induced tumor angiogenesis, and macrophage recruitment is inhibited in T^482^S^484^/AA kindlin-3 cells; Twist expression was significantly blunted in vitro and in tumors derived T^482^S^484^/AA kindlin-3–expressing cells; kindlin-3–mediated regulation of the EMT program in BC depends on kindlin-3 phosphorylation. Collectively, our findings identify kindlin-3 phosphorylation as a novel and key mechanism of tumor promoter function of kindlin-3 in BC.

## Materials and Methods

### Antibodies and reagents

Mouse mAb against EGFP (B2) was from Santa Cruz Biotechnology; rat mAb against CD29 (Mab 13) and rat mAb against mouse CD31 (MEC 13.3) were from BD Pharmingen; mouse mAb specific for activated human β_1_ integrin (HUTS-4) was from Millipore Sigma; mouse mAb against GAPDH (2D4A7) was from Novus Biologicals; rabbit mAb against actin (D18C11), rabbit mAb against Snail (C15D3), rabbit mAb to Src (36D10), and phosphor-Src (D49G4) were from Cell Signaling Technology; rat mAb anti-F4/80, Alexa-coupled secondary antibodies, and Alexa-coupled phalloidins were from Thermo Fisher Scientific; rabbit polyclonal antibodies against fibronectin were from Millipore Sigma; rabbit polyclonal antibodies against N-cadherin were from Thermo Fisher Scientific; rabbit polyclonal antibodies to Twist were from GeneTex; rabbit polyclonal antibodies against vWf were from Agilent; horseradish peroxidase–conjugated secondary antibodies were from Bio-Rad; and PKC inhibitors used were from MilliporeSigma: the PKC_α/β_ inhibitor was N-Myr-FARKGALRQ-NH_2_, the PKC_θ/δ_ inhibitor was 5-(3,4-Dimethoxyphenyl)-4-(1H-indol-5-ylamino)-3pyridinecarbonitrile, and the PKC_θ_ inhibitor was Myr-LHQRRGAIKQAKVHHVKC-NH_2_. ECL reagent was from Roche, RPMI, DMEM/F12, penicillin/streptomycin, and L-glutamine were from the Media Lab (Cleveland Clinic).

### cDNA constructs

EGFP-tagged kindlin-3 was created by cloning full-length kindlin-3 in a frame with EGFP into pEGFP-C2 vector (Clontech) as previously described (Malinin et al, 2009). All indicated mutations of kindlin-3 were introduced into constructs using QuikChange site-directed mutagenesis kits from Agilent Technologies (Santa Clara) and authenticated by DNA sequencing.

### Cell culture and transfections

Human MDA-MB-231 and BT549 BC lines and HEL cells were obtained from the American Type Culture Collection (ATCC) and cultured as per the ATTC instructions. Growth and morphology of the cells were routinely monitored, and they maintained key features consistent with prior descriptions of the lines. Derivative clones of the MDA-MB-231 cells with stable expression of EGFP and EGFP-kindlin-3 fusion proteins were established by nucleofection of EGFP-C2 plasmids expressing either EGFP alone or EGFP-kindlin-3 fusion constructs. Stable and continuous expression of the EGFP derivatives was maintained by growing cells in the presence of neomycin (1 mg/ml), and enrichment for the EGFP-expressing cells was obtained by FACS. Alterations in MDA-MB-231 and BT549 cell proliferation were determined by counting and comparing the number of viable cells over a period of 5 d after seeding the same number of cells on d 1. None of the expressed fusion proteins affected proliferation rates during this monitoring period.

### CRISPR/Cas9 gene editing–mediated targeting of kindlin-3 in BT549 cells

LentiCRISPRv2 lentiviral plasmid system (Addgene) was used to knockout kindlin-3 in BT549 BC cells using the protocol described by Cong and Zhang (2015). The human kindlin-3–specific sgRNAs were identified on the basis of two different predictive algorithms (Chopchop; https://chopchop.rc.fas.harvard.edu, and CRISPR Design, http://crispr.mit.edu), and the sgRNAs common to both algorithms were validated against human GECKOv2 sgRNA library. Only sgRNAs found in the GECKOv2 library were selected (Cong & Zhang, 2015). sgRNA oligos were purchased from Integrated DNA Technologies and subcloned into the lentiCRISPRv2 plasmid (Cong & Zhang, 2015). Lentivirus production and cell infections were performed as described previously (Taylor et al, 2013a, 2013b).

### Tissue and cell immunostaining

Tumor and lung tissues were collected after 9 or 12 wk after tumor implantation, snap-frozen in optimal cutting temperature medium (Sakura Finetek), and 8-μm sections were prepared. Blood vessels were visualized with rat antimouse CD31 followed by goat anti-rat Alexa Fluor 568 conjugate or with rabbit anti-vWF. Tumor sections were also stained with the following antibodies: rabbit anti-fibronectin and rabbit anti–N-cadherin followed by Alexa-568–conjugated goat antirabbit IgG. Stained sections were analyzed using fluorescent imaging microscopy (Leica) and ImageJ software. Positive staining areas were quantified in 10–15 independent fields/section.

HEL cells were stimulated with 800 nM PMA and plated onto fibrinogen-coated (20 μg/ml) glass slides for the times indicated, fixed with 4% PFA, permeabilized with 0.1% Triton X-100, blocked in horse serum, and stained with the specified antibodies for ∼18 h. BT549 cells were spread on 20 μg/ml of fibronectin and processed as described for HEL cells. Antigen–antibody complexes were detected by staining with Alexa-coupled secondary antibodies for 1 h. Stained cells were visualized with a 40× or 63× 1.4 oil objectives using a Leica TCS-NT laser scanning confocal microscope (Imaging Core; Cleveland Clinic). Laser intensities were adjusted to eliminate cross-over between channels, and the images were collected using Leica Confocal Software (version 2.5 Build 1227).

### Invasion assays

For invasion assays, modified Boyden chambers were coated with Matrigel (1:1 dilution; BD Biosciences) and used to compare the invasiveness of MDA-MB-231 cells expressing EGFP alone or EGFP–kindlin-3 fusion proteins in response to 10% serum as described (Sossey-Alaoui et al, 2014). The invading cells were stained with Alexa-488 phalloidin and analyzed using fluorescent imaging microscopy (Leica) and ImageJ software. Cell number was quantified in 10–15 independent fields/chamber.

### VEGF ELISA assay

VEGF secreted into the conditioned medium of MDA-MDB231 cells was quantified using the ELISA development kit and polyclonal rabbit antihuman VEGF from PeproTech as previously described (Sossey-Alaoui et al, 2014).

### Flow cytometry and soluble ligand binding

To assess β1 integrin activation, HUTS-4 binding to the different kindlin-3 transfectants was analyzed by gating only on the EGFP-positive cells. Mean fluorescence intensities (MFI) of mAb HUTS-4 binding were normalized to total β1 integrin surface expression measured with mAb 13. Cells (2 × 10^5^ cells) were incubated with HUTS-4 (20 μl) four in HBSS buffer containing 0.1% BSA, 0.5 mM CaCl_2_, MgCl_2_ for 30 min, and then fixed with 1% PFA for 10 min in room temperature. After washing, the cells were incubated with R-PE–labeled secondary antibody (10 μg/ml) for 30 min. Antibody binding was analyzed using LSRFortessa flow cytometer and FlowJo software (BD Biosciences).

### Development of kindlin-3 phosphoantibodies

C-LSLQRTGpSGG, C-LSLQRpTGSGG, and C-LSLQRTGSGG peptides were synthetized and purified in Molecular Biotechnology Core of Cleveland Clinic. Peptides were conjugation to KLH with glutaraldehyde. Four mice were injected into the peritoneum with an emulsion containing the antigen and an equal volume of Freund’s adjuvant. Complete Freund’s adjuvant was used for the first injection, Incomplete Freund’s was used for subsequent boosts, and no adjuvant was used for the final injection. Mice were injected at 3-wk intervals. 10 d after the third injection, the concentration of antibodies specific to each antigen was measured in the serum by ELISA. Spleen cells from the highest titer mouse were fused with SP2/0 cells by a standard PEG/DMSO method, and fused cells were selected using HAT media. 14 d later, supernatants were assayed by ELISA on the specific antigens. Cells from positive wells were expanded for cloning and individual cells producing antibodies were isolated from the mixed culture by the limiting dilution. Antibodies were developed in the Hybridoma Core of Cleveland Clinic. Antibodies were purified from tissue culture supernatants using Pierce Protein A IgG Purification Kit from Thermo Fisher Scientific.

### ELISA assay

50 μl of peptide (4 μg/ml) in coating buffer was added to individual wells of microtiter plates and incubated overnight at 4°C. The wells were washed three times with 100 μl PBS and 0.05% Tween 20, blocked with 150 μl of PBS containing 0.4% BSA, and incubated for 60 min in 37°C. Wells were washed three times with 100 ml PBS and 0.05% Tween 20, 50 μl of diluted antibody was added to each well, and the wells were incubated for 30 min in 37°C. After washing, 50 μl of secondary antibody was added to each well (rat-antimouse IgG1 from Southern Biotech). Reaction was developed by adding 100 μl of *o*-phenylenediamine dihydrochloride (Millipore Sigma), and incubation was carried out for 15 min. After sufficient color development, the reaction was terminated with 100 μl of 5N H_2_SO_4_. Absorbance was read at 492 nm.

### Platelet isolation

Platelets were isolated as described previously (Plow & Ginsberg, 1981) from the blood of healthy volunteers drawn into acid/citrate/dextrose, pH 4.6, containing 2 μM prostaglandin E_1_. Blood was centrifuged to obtain platelet-rich plasma, which was then further centrifuged, and the platelet pellet was suspended in Ca^2+^- and Mg^2+^-free Tyrode’s buffer (138 mM NaCl, 12 mM NaHCO_3_, 0.36 mM Na_2_HPO_4_, 2.9 mM KCl, 10 mM Hepes, 0.1% glucose, 0.1% bovine serum albumin, pH 7.1) and gel-filtered through a Sepharose CL-4B (GE Healthcare) column in Tyrode’s buffer to obtain the platelet preparations. Platelet counts were determined using a hemocytometer.

### Platelet aggregation and kindlin-3 phosphorylation

Effect of thrombin or PMA on kindlin-3 phosphorylation was examined using a kindlin-3 phosphospecific mAb while monitoring aggregation in an aggregometer (Chrono-log Corporation). Platelets (3 × 10^8^/ml) were equilibrated at 37°C for 5 min. Then, an agonist was added and aggregation was monitored for times indicated at 37°C with stirring. The final volume in each cuvette was 500 μl. Platelets were solubilized in a Laemmli buffer containing 62.5 mM Tris–HCl, pH 7.4, 2% SDS, 5% 2-mercaptoethanol, and 10% glycerol and subjected to SDS–PAGE. Proteins were transferred to PVDF, and blots probed with antibodies of interest using 5% BSA as a blocking agent.

### Isolation of mouse megakaryocytes and α_IIb_β_3_ activation assays

Bone marrow cells were harvested from 8- to 9-wk-old WT and kindlin-3 hypomorphic mice (Xu et al, 2014; Meller et al, 2017) by flushing femurs with Catch Buffer (PBS pH 7.4, 2% BSA, 0.38% trisodium citrate, DNAse I (1 U/ml), penicillin/streptomycin (100 U/ml). Mononuclear cells were isolated by centrifugation over Ficoll Hypaque Premium (1.080 g/ml; GE Healthcare) at 400*g* for 30 min at 22°C. Low-density mononuclear cells were cultured for up to 5 d at a starting density of 1 × 10^6^/ml in serum-free IMDM medium supplemented as described (Zauli et al, 1997; Shiraga et al, 1999) in the presence of 50 ng/ml murine TPO (R&D Systems) and 10 ng/ml murine IL-11 and IL-6 (Millipore Sigma). After 5 d in culture, the cells were treated with lentiviral vectors (Xu et al, 2018) expressing EGFP-tagged kindlin-3 (WT), QW/AA, TS/AA, or EGFP only as described. 48 h post-transduction, the cells were either untreated or stimulated with PAR-4 agonist peptide (100 μg/ml; Bachem) for 30 min at 37°C. α_IIb_β_3_ integrin activation was measured by flow cytometry of EGFP-positive cell populations using PE-labeled Ab to activation-dependent epitope of CD41/CD61 (clone JON/A) (Emfret Analytics) and rat antimouse total CD41-PerCP/Cy5.5 (BioLegend). The data are expressed as % activation = (MFI JONA/MFI total CD41) × 100.

### Immunoprecipitation

For antibody-mediated precipitation of endogenous kindlin-3, HEL cells were stimulated with PMA, and the samples were lysed in 50 mM Tris–HCl, pH 7.4, 150 mM NaCl chloride, 1% NP-40, and 1 mM CaCl_2_, containing protease and phosphatase inhibitors. Lysates were held on ice for 30 min before centrifugation at 12,000*g* for 15 min. Aliquots of the detergent-soluble material were precleared on A/G protein agarose for 1 h in 4°C. The lysates were incubated with 2 μl of antibodies or pre-immune serum and protein A/G agarose for 16 h at 4°C. Immunoprecipitated proteins were solubilized in Laemmli buffer and analyzed on Western blots with selected antibodies.

### Statistical analysis

Means and standard errors are reported. Statistical analyses using a two-tailed *t* tests were performed in SigmaPlot (version 11). Differences were considered to be significant with *P* < 0.05.

## Supplementary Material

Reviewer comments
