# Supplementary material for: Site-specific phosphorylation regulates the functions of kindlin-3 in a variety of cells
Source: Life Sci Alliance. 2020 Feb 5;3(3):e201900594. doi: 10.26508/lsa.201900594 (PMC7010036; doi:10.26508/lsa.201900594)
Supplement: Reviewer comments [file LSA-2019-00594_review_history.pdf]

A Site Specific Phosphorylation Regulates the Functions of Kindlin-3 in a Variety of Cells

Katarzyna Bialkowska, Khalid Sossey-Alaoui, Elzbieta Pluskota, Lahoucine Izem, Jun Qin and Edward F. Plow
DOI: 10.26508/lsa.201900594

Corresponding author(s): Dr. Edward F. Plow (Lerner Research Institute)

Review timeline:

Submission Date:	2019-10-30
Editorial Decision:	2019-10-31
Revision Received:	2020-01-06
Editorial Decision:	2020-01-09
Revision Received:	2020-01-22
Accepted:	2020-01-23

Scientific Editor: Andrea Leibfried

Transaction Report:

(Note: With the exception of the correction of typographical or spelling errors that could be a source of ambiguity, letters and reports are not edited. The original formatting of letters and referee reports may not be reflected in this compilation.)

Please note that the manuscript was previously reviewed at another journal and the reports were taken into account in the decision-making process at Life Science Alliance.

No full Peer Review Process File is available with this article, as the authors have chosen not to make the review process public in this case.

1st Editorial Decision 31 October 2019

October 31, 2019

Re: Life Science Alliance manuscript #LSA-2019-00594-T

Dr. Edward F. Plow
Lerner Research Institute
Department of Molecular Cardiology
Lerner Research Institute / NB50
9500 Euclid Avenue
Cleveland, USA-Cleveland, OH 44195 44195

Dear Dr. Plow,

Thank you for submitting your manuscript entitled "A Site Specific Phosphorylation Regulates the Functions of Kindlin-3 in a Variety of Cells" to Life Science Alliance. The manuscript was assessed by expert reviewers at another journal, and the editors transferred those reports to us with your permission.

The reviewers appreciated the robustness of the data provided, but would have expected a further reaching conceptual advance. We would like to invite you to submit a revised version of your manuscript for publication in Life Science Alliance. We would expect you to address the minor points of reviewer #3 (requested labelling and quantifications). We would also like to encourage you to add some experimental data to address the other points mentioned by this reviewer - maybe you have data already at hand to do so. We think this would further strengthen the work and increase its value to the field.

To upload the revised version of your manuscript, please log in to your account: https://lsa.msubmit.net/cgi-bin/main.plex
You will be guided to complete the submission of your revised manuscript and to fill in all necessary information. Please get in touch in case you do not know or remember your login name.

We would be happy to discuss the individual revision points further with you should this be helpful.

While you are revising your manuscript, please also attend to the below editorial points to help expedite the publication of your manuscript. Please direct any editorial questions to the journal office.

When submitting the revision, please include a letter addressing the reviewers' comments point by point.

Thank you for this interesting contribution to Life Science Alliance. We are looking forward to receiving your revised manuscript.

Sincerely,

Andrea Leibfried, PhD
Executive Editor
Life Science Alliance
Meyerhofstr. 1

69117 Heidelberg, Germany
t +49 6221 8891 502
e a.leibfried@life-science-alliance.org
www.life-science-alliance.org

A. THESE ITEMS ARE REQUIRED FOR REVISIONS

-- A letter addressing the reviewers' comments point by point.

-- An editable version of the final text (.DOC or .DOCX) is needed for copyediting (no PDFs).

-- High-resolution figure, supplementary figure and video files uploaded as individual files: See our detailed guidelines for preparing your production-ready images, http://www.life-science-alliance.org/authors

-- Summary blurb (enter in submission system): A short text summarizing in a single sentence the study (max. 200 characters including spaces). This text is used in conjunction with the titles of papers, hence should be informative and complementary to the title and running title. It should describe the context and significance of the findings for a general readership; it should be written in the present tense and refer to the work in the third person. Author names should not be mentioned.

B. MANUSCRIPT ORGANIZATION AND FORMATTING:

Full guidelines are available on our Instructions for Authors page, http://www.life-science-alliance.org/authors

We encourage our authors to provide original source data, particularly uncropped/-processed electrophoretic blots and spreadsheets for the main figures of the manuscript. If you would like to add source data, we would welcome one PDF/Excel-file per figure for this information. These files will be linked online as supplementary "Source Data" files.

IMPORTANT: It is Life Science Alliance policy that if requested, original data images must be made available. Failure to provide original images upon request will result in unavoidable delays in publication. Please ensure that you have access to all original microscopy and blot data images before submitting your revision.

2nd Editorial Decision 09 January 2020

January 9, 2020

RE: Life Science Alliance Manuscript #LSA-2019-00594-TR

Dr. Edward F. Plow
Lerner Research Institute
Department of Molecular Cardiology
Lerner Research Institute / NB50
9500 Euclid Avenue
Cleveland, USA-Cleveland, OH 44195 44195

Dear Dr. Plow,

Thank you for submitting your revised manuscript entitled "A Site Specific Phosphorylation Regulates the Functions of Kindlin-3 in a Variety of Cells". I have now assessed the changes introduced in response to the requests the editors of Life Science Alliance made in light of the reviewer reports at hand.

We would be happy to publish your paper in Life Science Alliance pending final minor revisions. I think the information provided in the figure panels still needs some improvement: please mention inhibitor in Figure 1C to avoid mis-interpretation. Please also improve the labelling in Figure 4A and 5A.

Furthermore:
- Please state in the figure legends "EGFP-kindlin3-overexpression" instead of "kindlin-3-overexpression" to allow easier interpretation of the figures
- Please make sure to mention the number of replicates throughout the manuscript (in either M&M section or in the figure legends)
- Please mention the statistical test performed next to the p-value mentioned in the figure legends
- Please note that we adhere to ICMJE authorship guidelines - please check all author contributions again
- Please add scale bars to figures 5B and C
- Please link your profile in our submission system to your ORCID iD - you should have received an email with instructions on how to do so

If you are planning a press release on your work, please inform us immediately to allow informing our production team and scheduling a release date.

To upload the final version of your manuscript, please log in to your account: https://lsa.msubmit.net/cgi-bin/main.plex
You will be guided to complete the submission of your revised manuscript and to fill in all necessary information. Please get in touch in case you do not know or remember your login name.

To avoid unnecessary delays in the acceptance and publication of your paper, please read the following information carefully.

A. FINAL FILES:

These items are required for acceptance.

-- An editable version of the final text (.DOC or .DOCX) is needed for copyediting (no

PDFs).

-- High-resolution figure, supplementary figure and video files uploaded as individual files: See our detailed guidelines for preparing your production-ready images, http://www.life-science-alliance.org/authors

-- Summary blurb (enter in submission system): A short text summarizing in a single sentence the study (max. 200 characters including spaces). This text is used in conjunction with the titles of papers, hence should be informative and complementary to the title. It should describe the context and significance of the findings for a general readership; it should be written in the present tense and refer to the work in the third person. Author names should not be mentioned.

B. MANUSCRIPT ORGANIZATION AND FORMATTING:

Full guidelines are available on our Instructions for Authors page, http://www.life-science-alliance.org/authors

We encourage our authors to provide original source data, particularly uncropped/-processed electrophoretic blots and spreadsheets for the main figures of the manuscript. If you would like to add source data, we would welcome one PDF/Excel-file per figure for this information. These files will be linked online as supplementary "Source Data" files.

Submission of a paper that does not conform to Life Science Alliance guidelines will delay the acceptance of your manuscript.

It is Life Science Alliance policy that if requested, original data images must be made available to the editors. Failure to provide original images upon request will result in unavoidable delays in publication. Please ensure that you have access to all original data images prior to final submission.

The license to publish form must be signed before your manuscript can be sent to production. A link to the electronic license to publish form will be sent to the corresponding author only. Please take a moment to check your funder requirements.

Reviews, decision letters, and point-by-point responses associated with peer-review at Life Science Alliance will be published online, alongside the manuscript. If you do want to opt out of having the reviewer reports and your point-by-point responses displayed, please let us know immediately.

Thank you for your attention to these final processing requirements. Please revise and format the manuscript and upload materials within 7 days.

Thank you for this interesting contribution, we look forward to publishing your paper in Life Science Alliance.

Sincerely,

Andrea Leibfried, PhD
Executive Editor
Life Science Alliance
Meyerhofstr. 1
69117 Heidelberg, Germany
t +49 6221 8891 502
e a.leibfried@life-science-alliance.org

www.life-science-alliance.org

3rd Editorial Decision 23 January 2020

January 23, 2020

RE: Life Science Alliance Manuscript #LSA-2019-00594-TRR

Dr. Edward F. Plow
Lerner Research Institute
Department of Molecular Cardiology
Lerner Research Institute / NB50
9500 Euclid Avenue
Cleveland, USA-Cleveland, OH 44195 44195

Dear Dr. Plow,

Thank you for submitting your Research Article entitled "A Site Specific Phosphorylation Regulates the Functions of Kindlin-3 in a Variety of Cells". It is a pleasure to let you know that your manuscript is now accepted for publication in Life Science Alliance. Congratulations on this interesting work.

The final published version of your manuscript will be deposited by us to PubMed Central upon online publication.

Your manuscript will now progress through copyediting and proofing. It is journal policy that authors provide original data upon request.

Reviews, decision letters, and point-by-point responses associated with peer-review at Life Science Alliance will be published online, alongside the manuscript. If you do want to opt out of having the reviewer reports and your point-by-point responses displayed, please let us know immediately.

IMPORTANT: If you will be unreachable at any time, please provide us with the email address of an alternate author. Failure to respond to routine queries may lead to unavoidable delays in publication.

Scheduling details will be available from our production department. You will receive proofs shortly before the publication date. Only essential corrections can be made at the proof stage so if there are any minor final changes you wish to make to the manuscript, please let the journal office know now.

DISTRIBUTION OF MATERIALS:
Authors are required to distribute freely any materials used in experiments published in Life Science Alliance. Authors are encouraged to deposit materials used in their studies to the appropriate repositories for distribution to researchers.

You can contact the journal office with any questions, contact@life-science-alliance.org

Again, congratulations on a very nice paper. I hope you found the review process to be constructive and are pleased with how the manuscript was handled editorially. We look forward to future exciting submissions from your lab.

Sincerely,

Andrea Leibfried, PhD
Executive Editor

Life Science Alliance
Meyerhofstr. 1
69117 Heidelberg, Germany
t +49 6221 8891 502
e a.leibfried@life-science-alliance.org
www.life-science-alliance.org
